# Interatrial block, P terminal force or fragmented QRS do not predict new-onset atrial fibrillation in patients with severe chronic kidney disease

**DOI:** 10.1186/s12872-020-01719-3

**Published:** 2020-10-07

**Authors:** Tapio Hellman, Markus Hakamäki, Roosa Lankinen, Niina Koivuviita, Jussi Pärkkä, Petri Kallio, Tuomas Kiviniemi, K. E. Juhani Airaksinen, Mikko J. Järvisalo, Kaj Metsärinne

**Affiliations:** 1 Kidney Center, Turku University Hospital and University of Turku, Hämeentie 11, PO Box 52, 20521 Turku, Finland; 2 Department of Clinical Physiology, Turku University Hospital and University of Turku, Hämeentie 11, PO Box 52, 20521 Turku, Finland; 3 Paavo Nurmi Centre & Unit for Health and Physical Activity, University of Turku, Kiinamyllynkatu 10, 20520 Turku, Finland; 4 Heart Center, Turku University Hospital and University of Turku, Hämeentie 11, PO Box 52, 20521 Turku, Finland; 5 Department of Anaesthesiology and Intensive Care, Turku University Hospital and University of Turku, Hämeentie 11, PO Box 52, 20521 Turku, Finland; 6 Perioperative Services, Intensive Care and Pain Medicine, Turku University Hospital and University of Turku, Hämeentie 11, PO Box 52, 20521 Turku, Finland

**Keywords:** Atrial fibrillation, Chronic kidney disease, Left atrial enlargement, Fragmented QRS, Incidence

## Abstract

**Background:**

The prevalence of left atrial enlargement (LAE) and fragmented QRS (fQRS) diagnosed using ECG criteria in patients with severe chronic kidney disease (CKD) is unknown. Furthermore, there is limited data on predicting new-onset atrial fibrillation (AF) with LAE or fQRS in this patient group.

**Methods:**

We enrolled 165 consecutive non-dialysis patients with CKD stage 4–5 without prior AF diagnosis between 2013 and 2017 in a prospective follow-up cohort study. LAE was defined as total P-wave duration ≥120 ms in lead II ± > 1 biphasic P-waves in leads II, III or aVF; or duration of terminal negative portion of P-wave > 40 ms or depth of terminal negative portion of P-wave > 1 mm in lead V_1_ from a baseline ECG, respectively. fQRS was defined as the presence of a notched R or S wave or the presence of ≥1 additional R waves (R’) or; in the presence of a wide QRS complex (> 120 ms), > 2 notches in R or S waves in two contiguous leads corresponding to a myocardial region, respectively.

**Results:**

Mean age of the patients was 59 (SD 14) years, 56/165 (33.9%) were female and the mean estimated glomerular filtration rate was 12.8 ml/min/1.73m^2^. Altogether 29/165 (17.6%) patients were observed with new-onset AF within median follow-up of 3 [IQR 3, range 2–6] years. At baseline, 137/165 (83.0%) and 144/165 (87.3%) patients were observed with LAE and fQRS, respectively. Furthermore, LAE and fQRS co-existed in 121/165 (73.3%) patients. Neither findings were associated with the risk of new-onset AF within follow-up.

**Conclusion:**

The prevalence of LAE and fQRS at baseline in this study on CKD stage 4–5 patients not on dialysis was very high. However, LAE or fQRS failed to predict occurrence of new-onset AF in these patients.

## Background

Patients with chronic kidney disease (CKD) have a high prevalence and incidence of atrial fibrillation (AF), which further increases the already high risk for cardiovascular events as well as accelerated CKD progression in affected patients [1–3]. Several clinical risk factors for AF have been identified in CKD patients [4, 5]. While interatrial block (IAB), P-wave terminal force (PTF), and fragmented QRS (fQRS) have been linked to the incidence of new-onset AF in selected patient groups in previous studies, data on predicting AF with ECG biomarkers in patients with severe CKD is scarce [6–9].

Thus, we sought to investigate the prevalence of ECG characteristics reflecting the presence of left atrial enlargement (LAE) and fQRS as well as their predictive performance on new-onset AF in patients with CKD stage 4–5 in a prospective follow-up study.

## Methods

The Chronic Arterial Disease, quality of life and mortality in chronic KIDney injury (CADKID)-study (http://www.ClinicalTrials.gov NCT04223726) is a prospective follow-up cohort study assessing cardiovascular disease, quality of life, and mortality in patients with CKD stage 4–5. This paper is a prespecified report from the CADKID study.

The inclusion criteria for the CADKID-study were CKD stage 4–5 defined as estimated glomerular filtration rate (eGFR) < 30 ml/min per 1.73 m^2^ calculated using the Chronic Kidney Disease Epidemiology Collaboration (CKD-EPI) formula, age over 18 years and residency in the catchment area of the hospital district of South West Finland.

Altogether 210 consecutive patients referred to the predialysis outpatient clinic of the Kidney Center of Turku University Hospital in 2013–2017 were recruited for the main CADKID study. As the present report focuses on ECG characteristics in patients with severe CKD and AF prediction, 41 patients with prior AF diagnosis, 3 patients with new-onset AF observed in the baseline ECG and 1 patient with ventricular pacemaker rhythm in the baseline ECG were excluded. Thus, the study cohort comprised 165 patients.

Relevant medical history and medications at baseline as well as all follow-up data were manually collected from the patient registry by the researchers. The baseline laboratory tests and a 12-lead surface electrocardiogram (ECG) were gathered by the certified laboratory services of Turku University Hospital (TYKSLAB). The patients were regularly at least every three months followed-up and interviewed for AF related symptoms in the research hospital. ECGs or 24 h ECG recordings were collected from all symptomatic patients while regular follow-up ECGs were not recorded.

The ECG recording settings were set at the paper speed of 50 mm per second and the standardized voltage ratio of 1 mm per 1 mV. The total P-wave duration was assessed in the lead II, the duration and depth of the terminal negative portion of P-wave in lead V_1_ and the number of biphasic P-waves in the inferior leads II, III and aVF, respectively. All ECGs were manually assessed in pdf format and digital magnification up to 400% without diminishing image quality by the researchers.

**Fig. 1:**
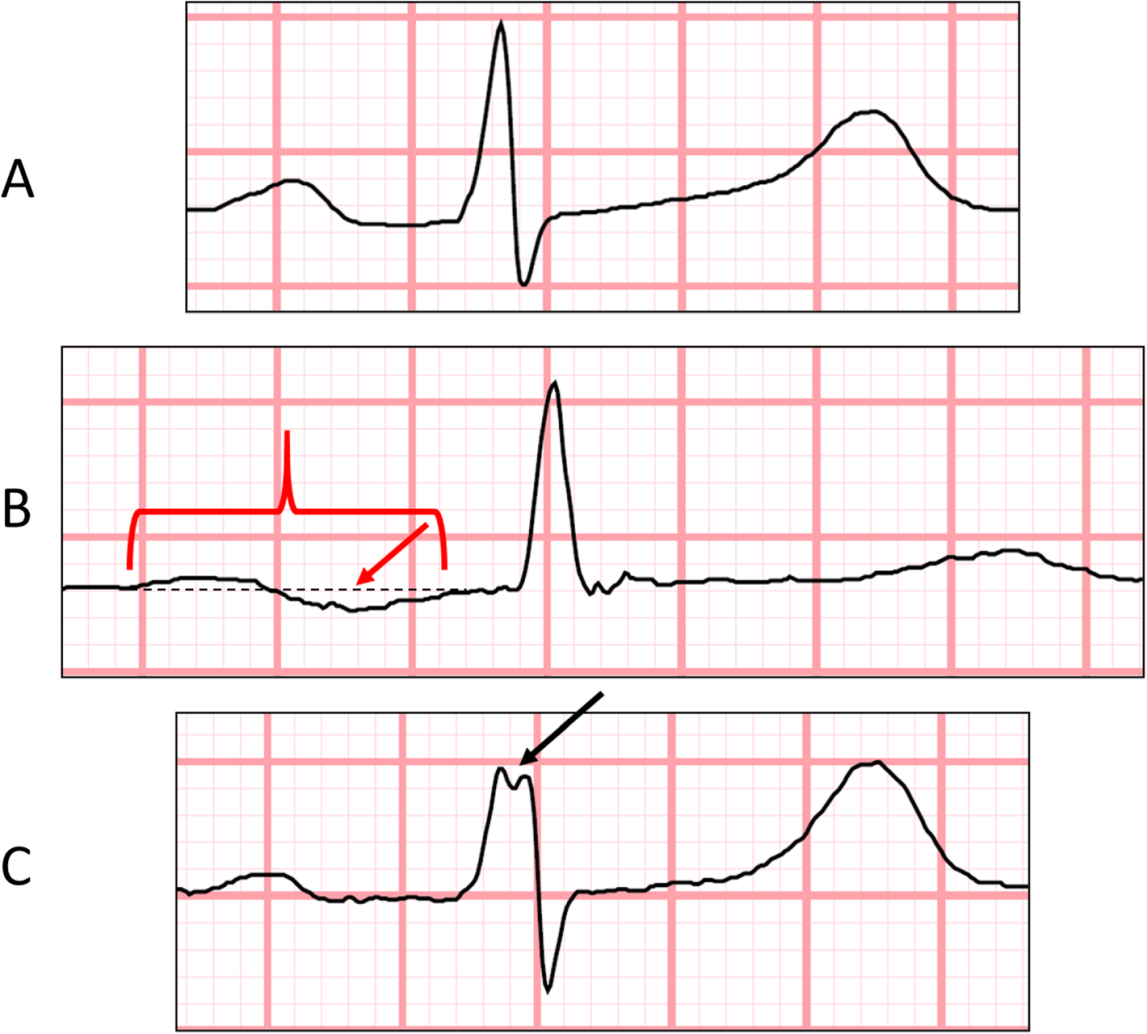
Presentation of an ECG with normal configuration, severe interatrial block and QRS complex fragmentation. Demonstration of ECGs with normal P-wave duration (< 120 ms) and normal QRS complex morphology in lead II (**a**), severe interatrial block with P-wave duration of 230 ms (brackets) and biphasic P-wave configuration (red arrow) in lead II (**b**) and fragmented QRS complex with notching observed in the R-wave (black arrow) in lead V3, respectively. The ECGs were recorded at rest at the paper speed of 50 mm per second and voltage ratio of 1 mm per 1 mV

The presence of LAE was defined according to following ECG criteria: total P-wave duration ≥120 ms measured in lead II (definition of first degree IAB) or P-wave duration ≥120 ms measured in lead II and more than one biphasic P-waves in leads II, III or aVF (definition of third degree IAB); or duration of terminal negative portion of P-wave > 40 ms measured in lead V_1_ or depth of terminal negative portion of P-wave > 1 mm measured in lead V_1_ or PTF > 0.04 mm*s (defined as the product of the duration and depth of the terminal negative portion of P-wave in lead V_1_) (Fig. 1). The presence of fQRS was defined according to ECG criteria: notching in the nadir of R or S wave or the presence of one or more additional R waves (R’) or, in the presence of a wide QRS complex (> 120 ms), more than two notches in R or S waves in at least two contiguous leads corresponding to a myocardial region with leads V_1_-V_4_, leads V_5_-V_6_ and I and aVL, and leads II-III and aVF denoting anterior, lateral and inferior myocardial regions, respectively [10] (Fig. 1).

The primary end-point of the study was the occurrence of new-onset AF confirmed by ECG or pacemaker log.

### Ethics

This study received approval by the Medical Ethics Committee of the Hospital District of Southwest Finland and adheres to the Declaration of Helsinki. Each participant provided written informed consent before study enrollment.

### Statistics

Normally distributed continuous covariates were reported as mean ± standard deviation (SD), skewed continuous variables as median [inter-quartile range (IQR)] and categorical covariates with absolute and relative (percentage) frequencies. Kolmogorov-Smirnov and Shapiro-Wilk tests were used to assess normality in continuous covariates. Continuous covariates and categorical covariates were compared using the unpaired t-test or Mann-Whitney test and Pearson × 2 or Fisher’s exact test, respectively. Baseline covariates correlating at *p* < 0.10 significance level with the dependent covariate in the univariate analysis were entered in the multivariate logistic regression analysis. All tests were two-sided and significance was set at *p* = 0.05. IBM SPSS Statistics software version 26.0 was used to perform all analyses.

## Results

Mean age of the study patients was 59 (SD 14) years and 56/165 (33.9%) were female. Altogether, 160/165 (97.0%) patients had a history of hypertension, 24/165 (14.5%) had heart failure, 71/165 (43.0%) had diabetes, and 17/165 (10.3%) had coronary artery disease (CAD), respectively. The mean eGFR was 12.8 ml/min/1.73m^2^. Overall 29/165 (17.6%) patients were diagnosed with new-onset AF during a median follow-up of 3 [IQR 3, range 2–6] years. The median time to occurrence of new-onset AF was 18 [IQR 26, range 2–66] months. Out of the 29 patients with new-onset AF, 25 (86.2%), 2 (6.9%) and 2 (6.9%) patients were diagnosed with paroxysmal AF, long-standing persistent AF (defined as AF episode lasting up to one year) and chronic AF, respectively.

**Fig. 2:**
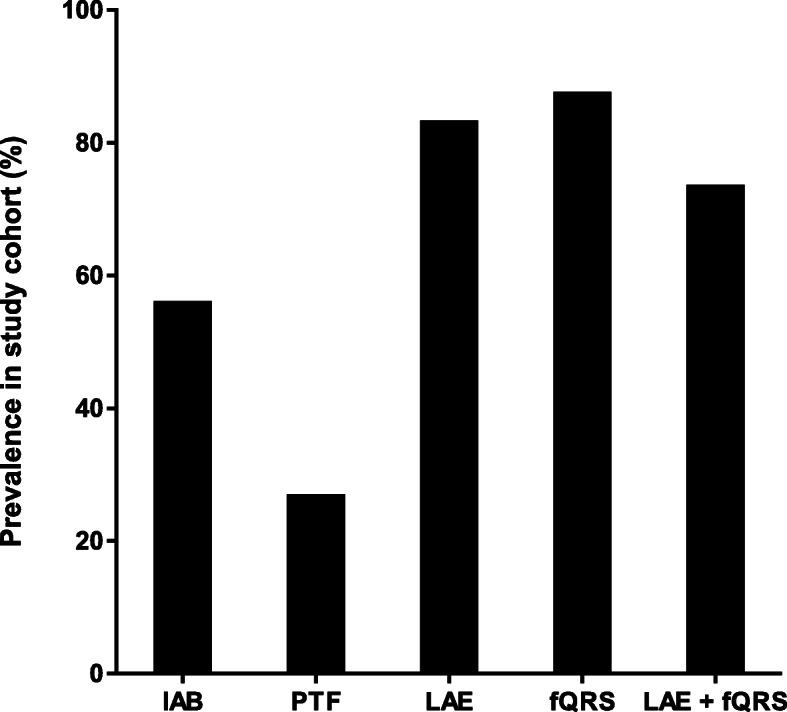
Prevalence of ECG markers in the study cohort. IAB = interatrial block; PTF = P-wave terminal force; LAE = left atrial enlargement; fQRS = fragmented QRS-complex

The ECG characteristics of patients according to the occurrence of new-onset AF are depicted in Table 1. Overall, 137/165 (83.0%) patients had LAE at baseline. Furthermore, IAB and PTF were present in 92/165 (55.8%) and 44/165 (26.7%) patients, respectively (Fig. 2). The patients with LAE were older and had longer QRS duration and higher N-terminal pro b-type natriuretic peptide (NT-ProBNP) in the univariate analysis. In the multivariate logistic regression analysis a QRS duration ≥100 ms (OR 4.20, CI95% 1.36–12.95, *p* = 0.01) independently predicted LAE in the baseline ECG. However, the presence of LAE was not associated with the incidence of new-onset AF.

**Table 1:** Electrocardiogram characteristics of patients according to incidence of new-onset AF during follow-up

	No AF (***N*** = 136)	Incident AF (***N*** = 29)	p
**ECG**			
**PR-interval**^**a**^			
**mean (median) ms**	176 (168)	180 (168)	0.48
**P-wave duration**^**a**^			
**mean (median) ms**	117 (120)	118 (120)	0.79
**Biphasic P-waves (> 1)**^**b**^	5 (3.7)	2 (6.9)	0.61
**Terminal P negativity depth**			
**mean (median) mV**^**c**^	0.5 (0.5)	0.6 (0.5)	0.39
**Terminal P negativity duration**			
**mean (median) mm**^**c**^	56 (50)	53 (60)	0.65
**IAB**^**d**^	75 (55.1)	17 (58.6)	0.84
PTF > 0.04 mm*s^**e**^	33 (24.3)	11 (37.9)	0.17
**LAE**^**f**^	113 (83.1)	24 (82.8)	1.0
**QRS duration mean (median) ms**	98 (95)	97 (96)	0.94
**QTc duration mean (median) ms**	442 (439)	426 (449)	0.44
**QRS axis mean (median) degrees**	25 (25)	15 (15)	0.15
**fQRS**^**g**^	121 (89.0)	23 (79.3)	0.22
**fQRS positive leads mean (median)**	5 (5)	4 (4)	0.07
**fQRS positive in anterior leads alone**^**h**^	5 (3.7)	2 (6.9)	0.61
**fQRS positive in lateral leads alone**^**i**^	9 (6.6)	0 (0)	0.36
**fQRS positive in inferior leads alone**^**j**^	31 (22.8)	8 (27.6)	0.63
**fQRS positive in ant + lat leads**	3 (2.2)	0 (0)	1.0
**fQRS positive in ant + inf leads**	20 (14.7)	5 (17.2)	0.78
**fQRS positive in lat + inf leads**	33 (24.3)	8 (27.6)	0.81
**fQRS positive in ant + lat + inf leads**	20 (14.7)	0 (0)	0.03
**Echocardiography**			
LA diameter mean (median) mm^*^	40 (39)	44 (42)	0.01
**EF mean (median) %**	65 (65)	65 (66)	0.22
**Laboratory tests**			
**NT-ProBNP median (IQR) ng/l**	808 (1632)	1390 (5064)	0.10
**Clinical charateristics**			
**CHA**_**2**_**DS**_**2**_**-VASc-score median (IQR)**	2 (2)	3 (3)	0.03

^*^data is missing in 28 (17.0%) cases

^a^measured in lead II; ^b^ measured in leads II, III and aVF; ^c^ measured in lead V_1_; ^d^ defined according to ECG-criteria (total P-wave duration ≥120 ms in lead II ± more than one biphasic P-waves in leads II, III or aVF); ^e^ defined according to ECG-criteria (the product of the duration (in seconds) and depth (in millimeters) of the terminal negative portion of P-wave in lead V_1_); ^f^ defined according to ECG-criteria (total P-wave duration ≥120 ms in lead II ± more than one biphasic P-waves in leads II, III or aVF; or duration of terminal negative portion of P-wave in lead V_1_ > 40 ms or depth of terminal negative portion of P-wave in lead V_1_ > 1 mm); ^g^ defined according to ECG-criteria (presence of a notched R or S wave or the presence of one or more additional R waves (R’) or, in the presence of a wide QRS complex (> 120 ms), more than two notches in R or S waves in two contiguous leads corresponding to a major coronary artery, respectively.); ^h^ measured in leads V_1_-V_4_; ^i^ measured in leads V_5_-V_6_ and I and aVL; ^j^ measured in leads II-III and aVF

Values in parentheses are % unless stated otherwise. ECG = electrocardiogram; IAB = interatrial block; PTF = P terminal force; LAE = left atrial enlargement; fQRS = fragmented QRS; LA = left atrium; EF = ejection fraction; NT-ProBNP = N-terminal pro b-type natriuretic peptide; IQR = inter-quartile range; CHA_2_DS_2_-VASc = congestive heart failure, hypertension, age ≥ 75 years (doubled), diabetes mellitus, prior stroke, transient ischemic attack or thromboembolism (doubled), vascular disease, age 65 to 74 years and sex category (female, unless < 65 years and no other risk factors)

Altogether 144/165 (87.3%) patients had fQRS in at least two contiguous leads in the baseline ECG. fQRS was observed in 55/165 (33.3%), 73/165 (44.2%), and 125/165 (75.8%) for anterior, lateral and inferior leads, respectively (Fig. 2). fQRS was present in multiple myocardial regions in 89/165 (53.9%) patients as well as in six of the nine patients with wide (> 120 ms) QRS complex. Younger age and absence of prior CAD diagnosis were associated with fQRS in the univariate analysis but no significant associations with fQRS were observed in the multivariate model. Although, the presence of fQRS in all myocardial regions in the baseline ECG was associated with a lower rate of new-onset AF in the univariate analysis (Table 1), overall, fQRS was not associated with the occurrence of new-onset AF. LAE and fQRS coexisted in 121/165 (73.3%) patients (Fig. 2).

The results considering AF prediction remained unchanged when the analyses were applied to patients who developed new-onset AF within one year or three years of follow-up or to those who developed new-onset long-standing persistent or chronic AF (data not shown).

## Discussion

This is the first study to assess the prevalence of both LAE and fQRS and their association with AF incidence in a large cohort of non-dialysis patients with CKD stage 4–5. The prevalence of, both, LAE and fQRS in the baseline ECG was remarkably high in this study. Furthermore, three quarters of patients were observed with both ECG patterns – a perception not demonstrated before in CKD patients. However, neither LAE nor fQRS were associated with the incidence of new-onset AF in this study.

The prevalence of IAB and PTF was high in our patients with severe CKD not on dialysis and the rate of both patterns was comparable to previous studies in hemodialysis patients [11, 12]. However, we found no connection between LAE and new-onset AF. The high prevalence of LAE in this study population probably better represents the gross overall cardiovascular disease burden in patients with CKD stage 4–5 than AF risk alone. In fact, both IAB and PTF have been associated with hypertension, CAD and heart failure which were highly prevalent conditions in our study cohort [13–15]. Both IAB and PTF have been described to reflect the presence of LAE and to predict the incidence of new-onset AF in various populations [6, 12, 16–18] but not in CKD patients.

Almost nine patients out of ten were observed with fQRS in our cohort – the highest prevalence reported to date. In previous studies on patients with established structural heart disease or AF, the prevalence of fQRS has ranged between 30 and 50% and the rates have been similar among patients with CKD stage 3–5 [8, 9, 19–21]. As with LAE, the high fQRS rate may partly be explained by the high rate of cardiovascular comorbidities in our study population since fQRS has been associated with diabetes, CAD and, vascular calcification in prior reports [8, 18, 22]. In recent studies, fQRS has been linked to the incidence of new-onset AF in patients with established heart disease, as well as to increased AF recurrence after cardioversion or catheter ablation [8, 9, 19, 23, 24]. It is unclear why no association between AF and fQRS was observed among patients with severe CKD in this study.

There is an unmet need for ECG markers of AF risk due to the high prevalence and often asymptomatic nature of AF among CKD patients. Moreover, incident stroke is often the first symptom of AF [25]. It is, therefore, disappointing that the ECG analysis in our study provided no predictors for AF in CKD patients. The recent advances in technology have brought forth new methods for detecting AF and accordingly, smart device applications have received plentiful attention [26]. Subsequently, older ECG based screening methods have also been newly approached. Recently a sophisticated artificial intelligence algorithm effectively predicted the incidence of AF from standard 12-lead ECGs at sinus rhythm [27]. While the novel approach fared well in a large non-CKD patient cohort, the performance of the algorithm is yet to be tested in CKD patients – especially in advanced CKD.

Patients with severe CKD, in addition to heavy cardiovascular disease burden, are at risk for vascular and tissue calcification [28] and while extensive vascular calcification may greatly increase the prevalence of LAE and fQRS, it may also “drown out” the predictive effect of these ECG biomarkers for AF. Furthermore, uremic toxins and dialysis may have precipitating effects for AF overshadowing ECG markers. Overall, non-dialysis patients with severe CKD appear to possess substantial substrate for AF, demonstrated by the annual AF incidence of 6% in our study, due to the high rate of co-existing conditions affecting the atria and ventricles assessed by LAE and fQRS. Furthermore, this clustering of risk factors may partly explain the strikingly high prevalence of the co-existence of LAE and fQRS in these patients. While these nonspecific ECG biomarkers possibly reflect high cardiovascular risk burden in CKD patients, they do not appear to predict AF in this population. While further research on ECG biomarkers in this setting is needed, other methods based on clinical risk assessment, biochemistry or echocardiography are likely to perform better in prediction of AF among these highly morbid patients – a matter to be addressed in future reports of the CADKID study.

### Limitations

This study has all the limitations of an observational study. The patient cohort was relatively small. However, all the study patients were extensively and consistently studied by the same trained researchers and quality of the data was high. While patients with severe CKD are at increased risk for electrolyte and fluid imbalance – known arrhythmogenic risk factors, conditions such as hyperkalemia or hypervolemia may have caused bias in the study. As AF is often asymptomatic, some self-limited new-onset AF episodes may have been missed. Nevertheless, all the study patients resided in the catchment area, were frequently and regularly, due to CKD severity, in contact with the Kidney Center and all symptomatic AF episodes are consistently recorded in the electronic archives of the research hospital. Despite these limitations, we believe that these data can benefit clinical practice and guide future research.

## Conclusions

Our study demonstrated a high prevalence of LAE and fQRS in patients with CKD stage 4–5. Our findings suggest that LAE or fQRS do not predict new-onset AF in these patients.

## Data Availability

The datasets used and/or analyzed during the current study available from the corresponding author on reasonable request.

## Abbreviations

CKD: Chronic kidney disease
AF: Atrial fibrillation
IAB: Interatrial block
PTF: P-wave terminal force
fQRS: Fragmented QRS
ECG: Electrocardiogram
LAE: Left atrial enlargement
CADKID-study: The chronic arterial disease quality of life and mortality in chronic KIDney injury study
eGFR: Estimated glomerular filtration rate
CKD-EPI formula: Chronic kidney disease epidemiology collaboration formula
TYKSLAB: The certified laboratory services of Turku University hospital
SD: Standard deviation
IQR: Inter-quartile range
CAD: Coronary artery disease
NT-ProBNP: N-terminal pro b-type natriuretic peptide
OR: Odds ratio
CI: Confidence interval
